# Comparison and Standardisation of Various Open Preperitoneal Techniques in Inguinal Hernia Surgery–Results of a Review and Consensus

**DOI:** 10.3389/jaws.2025.13990

**Published:** 2025-03-19

**Authors:** Ralph Lorenz, Willem Akkersdijk, Gabriel Paiva De Oliveira, Marc Soler, Jean-Francois Gillion, Augusto Lourenço, Rui Soares Da Costa, Edouard Pelissier, Franz Ugahary, Frederik Berrevoet

**Affiliations:** ^1^ Hernia Center 3+CHIRURGEN, Berlin, Germany; ^2^ Department of General and Abdominal Surgery, Clinic for General and Abdominal Surgery, Medical University Brandenburg an der Havel, Neuruppin, Germany; ^3^ Surgical Department, St Jansdal Hospital, Harderwijk, Netherlands; ^4^ Department for General Surgery, Hospital Garcia de Orta, Almada, Portugal; ^5^ Service de Chirurgie Viscérale et Digestive, Clinique Saint-Jean, Cagnes-sur-Mer, France; ^6^ Surgical Department, Ramsay Sante Hôpital Privé d’Antony, Antony, France; ^7^ Faculty of Healthcare Sciences, Beira Interior University, Covilhã, Portugal; ^8^ Department for General Surgery, Hospital Lusíadas Porto, Porto, Portugal; ^9^ Institut de la Hernie Paris, Paris, France; ^10^ Former General and Vascular Surgeon (NP), Ziekenhuis Rivierenland Tiel, Tiel, Netherlands; ^11^ Department of General and HPB Surgery and Liver Transplantation, Ghent University Hospital, Ghent, Belgium

**Keywords:** groin hernias, open preperitoneal techniques, MOPP, ONSTEP, TIPP, TREPP

## Abstract

**Introduction:**

Both open and laparoendoscopic preperitoneal mesh techniques are good options for the treatment of inguinal hernias. The 2023 updated HerniaSurge Guidelines recommend open preperitoneal mesh techniques as an acceptable alternative to Lichtenstein repair if a competent and experienced surgeon is available. However, although numerous open preperitoneal surgical techniques have been developed, only a few comparative studies comparing them are available. Because of the lack of scientific evidence and standardisation, the aim of this article is to define comparable standards and compare four frequently used open preperitoneal techniques.

**Method:**

Using a Delphi-consensus process among both the authors and experts in the field, various key steps for each procedure, indications, and outcome parameters were set to allow adequate comparison of different open preperitoneal techniques.

**Results:**

We present four different and frequently used open preperitoneal techniques: Minimal Open PrePeritoneal repair (MOPP), TransInguinal PrePeritoneal repair (TIPP), TransREctus sheat PrePeritoneal repair (TREPP), and Open New Simplifyed Total Extraperitoneal repair (ONSTEP). We provide a clear and comparable standard regarding the best indication, different procedural steps, the use of meshes and fixation, the learning curve involved, and possible complications and limitations. We also identify some similarities for the techniques but also specific differences on different topics.

**Conclusion:**

Development, validation, and implementation of these standards for the various open preperitoneal techniques are necessary both for education and training as well as for future comparative studies.

## Introduction

Inguinal hernias are one of the most common issues requiring surgical intervention worldwide. While previously there were only a few, mainly open surgical techniques without the use of synthetic meshes, numerous new surgical techniques have been developed in recent decades. The origins of open preperitoneal techniques can be traced back to the pioneering works of Stoppa, Nyhus, Read, and Wantz [1, 2]. Over time, various open surgical methods, both with and without the application of synthetic meshes, have been developed alongside advancements in endoscopic techniques. More recently, the use of surgical robots has also become an option for the treatment of inguinal hernias. To enable a meaningful scientific comparison of these methods, standardisation of surgical techniques is essential. Furthermore, standardise d approaches are critical for providing structured education and training in this field.

The guidelines for the treatment of inguinal hernias recommend a tailored approach depending on the patient’s characteristics, available resources, and the experience of the surgeons [3].

For the majority of inguinal hernias, mesh techniques are recommended, which can be performed both open and endoscopically [4]. Recent studies show that endoscopic techniques have advantages over the Lichtenstein technique in terms of chronic pain [4].

For endoscopic techniques, this standardisation has already been achieved over several publications [5–8]. For the Lichtenstein technique, a significant precision of the surgical technique was made decades ago with the Amid-modifications [9]. There have also been several recent publications on the Shouldice technique that aimed to standardise the procedure [10, 11].

Franz Ugahary is the founder of the modern minimally invasive and minimally open preperitoneal technique, developing the gridiron incision in 1995 [12].

The TIPP technique was developed in September 2004 by Edouard Pelissier after the first prosthesis specifically dedicated to being spread forward in the pre-peritoneal space was created: The Polysoft (©C.R.Bard) prosthesis [13, 14].

In 2005, A. Lourenco and R. S. da Costa from Porto developed the Onstep technique. Their goal was to simplify the procedure by placing the prosthesis partially in the preperitoneal space while simultaneously splitting it. This approach eliminated the need for the parietalization step, thereby making the technique easier to learn [15, 16].

In 2006, Willem Akkersdijk introduced the Trans Rectus Sheath PrePeritoneal (TREPP) technique [17, 18], building on the Ugahary technique and utilizing the TIPP (Pelissier) prosthesis. This method represents a precisely codified pure posterior approach, meticulously structured into nine distinct steps.

Building on the principles of Ugahary’s dissection and incorporating the steps of the TIPP technique, Marc Soler developed the MOPP technique. This method consistently places a preperitoneal mesh through the deep inguinal ring [12, 19, 20].

However, the diverse range of materials used in hernia surgery further complicates efforts toward standardisation.

Due to the lack of scientific literature and standardisation, this article aims to compile and summarise the essential key points of various open preperitoneal techniques. The goal is to establish a unified standard and provide a straightforward framework for comparing these techniques, serving as a foundation for future comparative studies.

## Methods

A systematic literature search was performed independently by the author’s steering group (RL, WA, GO, and MS) and reported on 1st July 2024. The Cochrane Library, PubMed, Embase, and Google Scholar were searched until 30th June 2024, using Medical Subject Heading (MeSH) terms “Open preperitoneal repair, groin hernia, TIPP, MOPP, TREPP, ONSTEP”. Records were screened by title and abstract for existing detailed procedure descriptions and technical standards of the following open preperitoneal techniques:

- MOPP = Minimal Open PrePeritoneal repair
- ONSTEP = Open New Simplified Total ExtraPeritoneal repair
- TIPP = Trans Inguinal PrePeritoneal repair
- TREPP = Trans REctus sheat PrePeritoneal Repair

The full texts were independently evaluated by the steering group. Only studies deemed acceptable or of high-quality according to the SIGN checklist were included to minimise the risk of bias. Any disagreements between assessors were resolved through group discussion. The steering group was selected based on their published research and expertise in inguinal hernia surgery.

An additional group of European surgeons experienced in open preperitoneal techniques and inguinal hernia repair (see Author list) discussed these findings from July 2024 to September 2024 to develop a consensus regarding standards of inguinal hernia repair.

**TABLE 1:** Comparison of four different open preperitoneal techniques.

	Question	MOPP	TIPP	TREPP	ONSTEP
1	Best or even ideal indication?	Primary groin hernias	Large direct or indirect and combined direct, indirect, and femoral hernias	Primary groin hernias	Non-obese men with small- and medium-size hernias (EHS Classification)
2	Skin incision location and length (Figure 1)	Groin transverse incision in front of the internal ring 3-4 cm	Groin transverse incision 4-5 cm along the inguinal canal, 1.5 cm above the pubic bone and, 1.5 cm lateral to the midline	Lower abdomen 5 cm transverse incision almost 2-3 cm above the inguinal canal	Lower abdomen 4 cm transverse incision almost 2-3 cm above the inguinal canal
3	Important preparation steps Use of specific instruments?	Always exact parietalisation to avoid overseen occult indirect hernias and to unroll the prosthesis			
		Different long Retractors (Figure 2)	Two Langenbeck or Kocher Retractor medial and lateral	Two Langenbeck or Kocher Retractor	One Langenbeck, Kocher, or Farabeuf Retractor (Figure 3)
4	Handling of the hernia sac or lipomas	Reducing hernia sac Resection of Lipomas	Resection of indirect hernia sac Reducing direct hernia sac Resection of lipomas	Reposition of indirect and direct hernia sac Resection of lipomas	Reposition of indirect and direct hernia sac Resection of lipomas
4	How to create preperitoneal space?	Blunt dissection with counted gauzes (one or two 10 × 10 cm gauzes)			
5	Mesh position	Complete preperitoneal mesh placement in Retzius space medially and Bogros space laterally			Medial: preperitoneal in the Retzius space Lateral: interparietal on top of the internal muscle (Figure 4)
		(Figure 5)	(Figure 6)	(Figure 7)	
6	How is access provided for mesh insertion in the groin?	Always *via* internal ring First medial placement than lateral placement of the mesh	Depending on type of hernia, indirect *via* internal ring or direct *via* posterior wall First medial placement than lateral placement of the mesh	Via opened rectus sheath First lateral placement then medial placement of the mesh	The medial part of the mesh is inserted in the preperitoneal space through an opening in the peri-tuberculum transversalis fascia after creating space with a gauze First medial placement than lateral placement of the mesh
7	Mesh size and type Preformed or flat?	Any type of preformed or flat lightweight mesh with large pores is recommended, with a minimum size of 8 × 14 cm. Meshes with a commercially resorbable recoil ring facilitate easier implantation. There appears to be no significant differences between various brands [21]			
		Non-split mesh			Split mesh: lateral to the internal ring surrounding spermatic cord or round ligament (Figure 8)
8	Is mesh fixation needed and, if so, how?	No fixation	Mostly no fixation, optional one or two non-resorbable single stitches as fixation on Cooper´s ligament to avoid mesh roll-up in case of large direct hernias	No fixation	No fixation is needed in ideal cases. A single Vicryl stitch to the pubic bone might provide benefits in women
9	Closure of the posterior wall – Augmentation or Bridging?	Normally no, optional augmentation with closure of the posterior wall	Normally no, optional augmentation with closure of the posterior wall	No	No
10	What are the limitations of the techniques?	Unsuitable for morbidly obese patients	Unsuitable for morbidly obese patients	Unsuitable for morbidly obese patients	Scrotal and femoral hernias
		For all techniques, hernia recurrences—especially after mesh repair or hernia repair following oncologic prostate resection with lymphadenectomy or vascular procedures—can present significant challenges			
11	Possible specific complications	For all techniques utilizing the preperitoneal space, complications in this area are possible, including injuries to the vessels (such as the inferior epigastric, iliac, or Corona mortis) or the bladder Recognition of perioperative vascular injury may not be straightforward postoperatively			
12	Average operating time (+ short <20′, ++ midterm 21′ to 40′, +++ longer >41′)	++	++	++	+
13	Learning curve of the technique (+short, ++ midterm, +++ longer)	++	++	+++ [22]	+

Using a modified Delphi methodology, the steering group identified the following four main domains of focus:

- Patient selection and indication (Table 1), Prehabilitation
- Technical steps of the preparation (Table 1)
- Technical steps of repair of the four different techniques (Table 1)
- Rehabilitation and aftercare

All authors were provided with a questionnaire regarding the individual techniques. First, the results of the independent questionnaires were compiled. Subsequently, statements were formulated by the steering group and then submitted for voting. In a final process, formulations were developed that achieved a minimum consensus of 75% among all authors.

## Results

We identified the following eight publications on the most important steps of the different procedures: MOPP, TIPP, TREPP, and ONSTEP [13–19, 23].

The steering group searched and summarised not only technical and procedural steps but also the specific use and fixation of meshes. We also incorporated best indications, limitations, potential complications, operating time, learning curves, and prehabilitation and rehabilitation protocols into the standard.

We concluded with a consensus on the four different and frequently used open preperitoneal techniques as a clear recommendation on how to do them as a standard.

All four techniques have numerous similarities:

- In experienced hands, addressing recurrences after previous anterior mesh or non-mesh repairs is feasible but can be particularly challenging, especially following prior mesh repairs using the Lichtenstein, Plug, or Gilbert techniques.
- Hyperextending the hip facilitates the preparation of the groin area, improving access and visibility during the procedure.
- Surgery under local anaesthesia is feasible for most techniques; however, general anaesthesia with a laryngeal mask is most commonly employed.
- All six layers of the abdominal wall should be identified (Skin, Camper´s fascia, Scarpa´s fascia, External oblique fascia, Internal oblique muscle, and Transversalis fascia).
- Hydrodissection with local anaesthesia is recommended for improved nerve identification and enhanced postoperative pain management.
- All nerves in the surgical area should always be systematically identified and, whenever feasible, preserved.
- All potential hernia defects (indirect, direct, or femoral) should always be systematically identified, and the exact parietalisation in the deep inguinal ring is mandatory.
- All hernias should be classified regarding the EHS-Classification into M, L, F, and C I, II, and III; Rx [24].
- In all cases, all potential preperitoneal lipomas should be identified and, preferably, excised.

**FIGURE 1:**
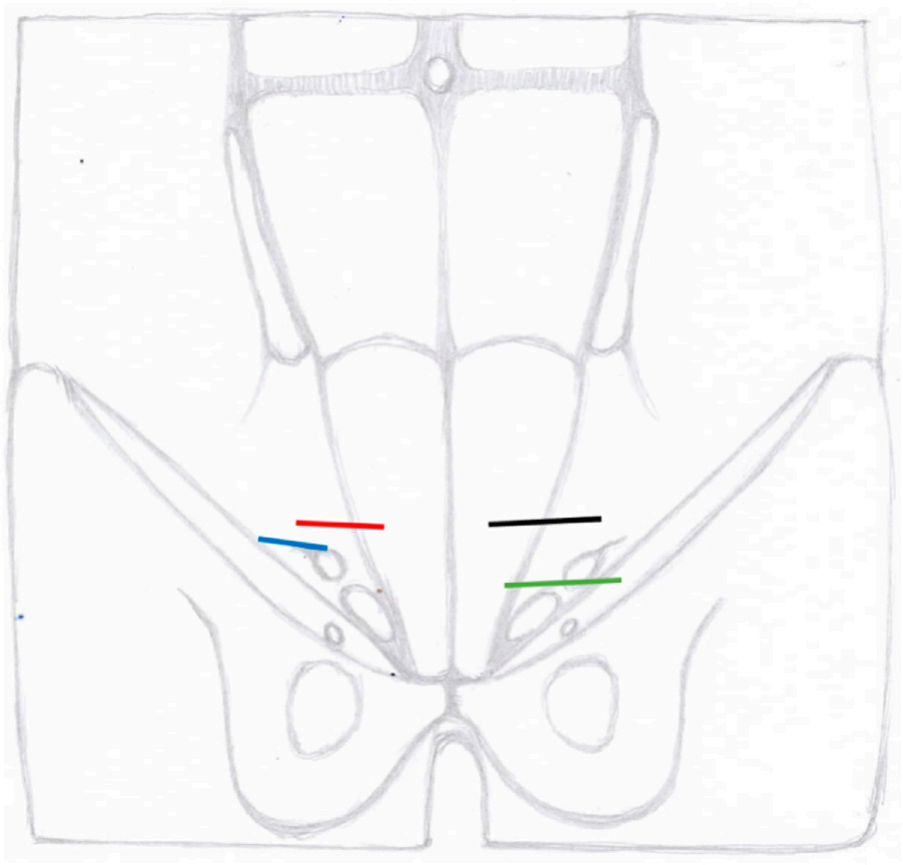
Localisation of skin incisions of different open preperitoneal techniques blue MOPP, red ONSTEP, green TIPP, black TREPP

**FIGURE 2:**
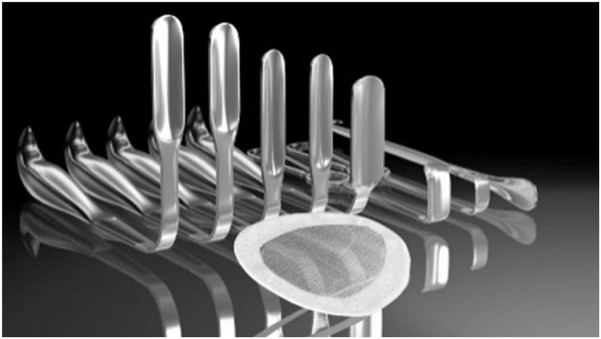
MOPP – Specific retractor instruments (^©^ M. Soler).

**FIGURE 3:**
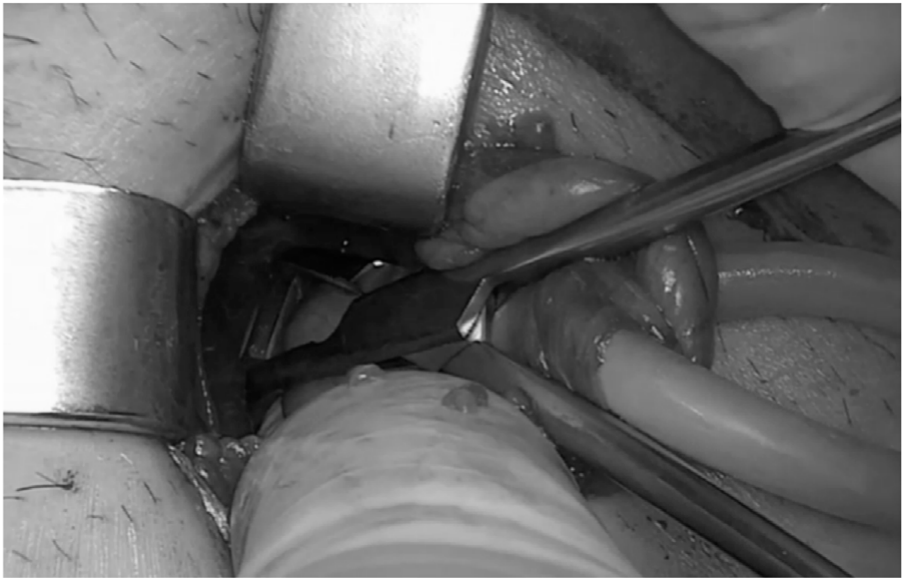
ONSTEP - Preparation of the Retzius space (^©^ G. Oliveira).

**FIGURE 4:**
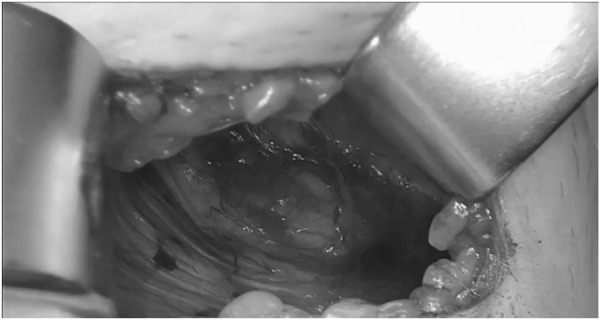
ONSTEP – View into the Retzius space (^©^ G. Oliveira).

**FIGURE 5:**
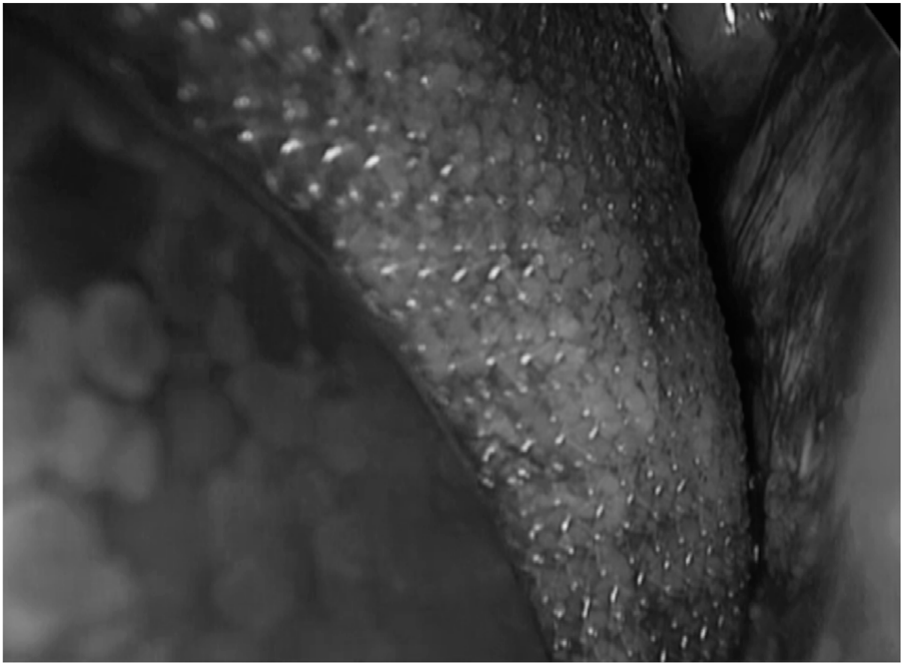
MOPP - Control of mesh position in the preperitoneal space (right Cooper’s ligament) (^©^ M. Soler).

**FIGURE 6:**
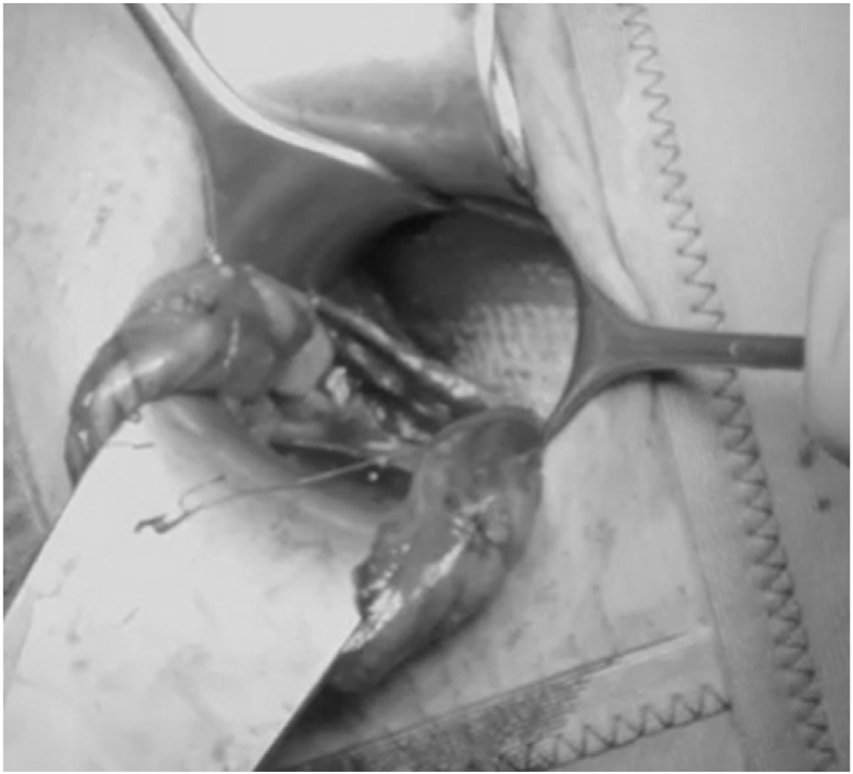
TIPP - Mesh position in the preperitoneal space (^©^ R. Lorenz).

**FIGURE 7:**
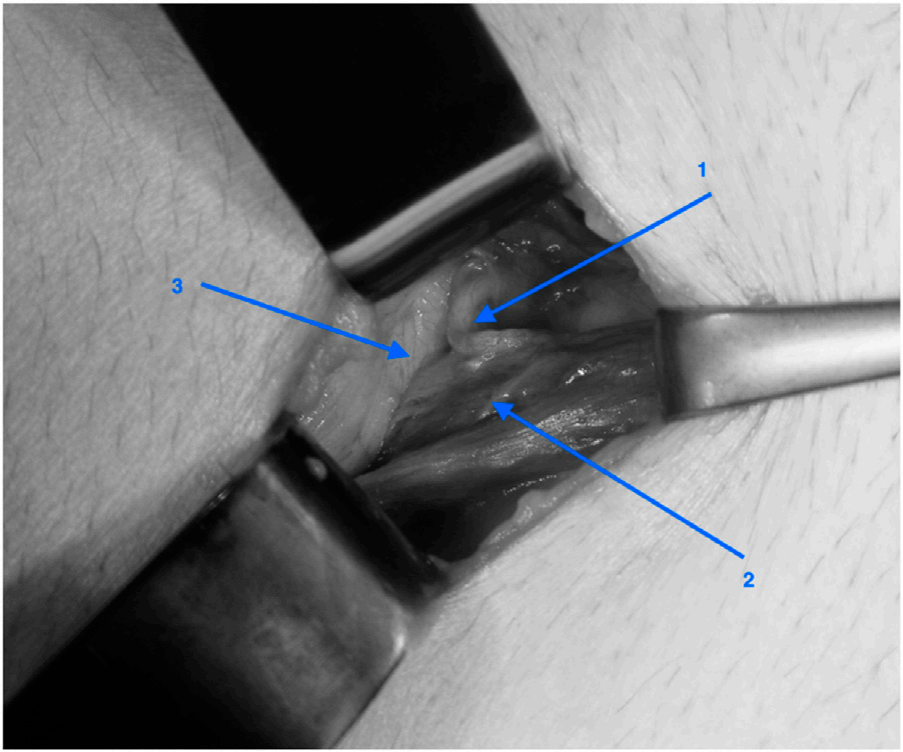
TREPP - Inspection of the cord after dissection of the preperitoneal space: 1: vas deferent, 2: a and v testicularis, 3: peritoneum (^©^ W. Akkersdijk).

**FIGURE 8:**
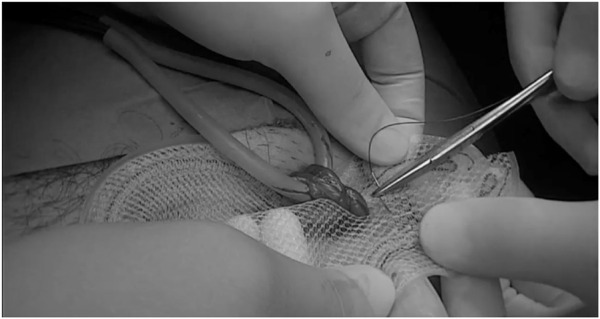
ONSTEP – Lateral mesh reconstruction (^©^ G. Oliveira).

The minimal or not necessary fixation of the meshes in the preperitoneal position seems to be a way to avoid acute and chronic postoperative pain [25].

As part of the prehabilitation, the authors recommend weight reduction and nicotine abstinence if possible. Single-shot antibiotics with cephalosporines are recommended only for high-risk patients according to the current updated HerniaSurge guidelines [4].

The rehabilitation begins intraoperatively with the use of local anaesthesia. After surgery, a therapy regimen could include proper pain medication, local cooling, and early mobilization. Pain-adapted physical rest is recommended during the first few weeks postoperatively. Return to normal work activities typically occurs within one to 2 weeks, while return to sport activities generally takes two to 3 weeks.

## Discussion

The laparo-endoscopic techniques TAPP and TEP are currently the gold standard for preperitoneal mesh repair of groin hernias.

The advantage of the open approach in inguinal hernia surgery primarily lies in the possibility of intraoperative tailoring based on the findings during the procedure. This allows the surgeon to adjust the surgical technique in real time, depending on the specific anatomical and pathological conditions encountered, thereby optimizing the outcome and minimizing complications. A disadvantage of open preperitoneal techniques is that they involve both the anterior and posterior planes of the groin.

In this study, we aimed to compare four common open preperitoneal techniques for the treatment of inguinal hernias. For each technique, there are existing publications outlining the key steps of the procedure. We have attempted to synchronize these key points in a simple, comparable format, and provide recommendations for a tailored approach.

However, there are other open preperitoneal techniques such as Usher, Nyhus Repair, Stoppa Repair, Rives Repair, Read Technique, Wantz Technique, Alexandre technique, Kugel Technique, Ugahary technique, and modified anterior preperitoneal repair = mAPP, that are less commonly performed today and differ from those mentioned in this analysis [26–29]. Fundamentally, all techniques share a common objective: to position a mesh within the preperitoneal space, ensuring effective coverage of the myopectineal orifice. This approach reinforces the abdominal wall and minimises the risk of hernia recurrence.

The differences between the techniques are minimal and primarily involve factors such as the location of entry into the preperitoneal space, the instruments required, the use of mesh fixation, the type of mesh, any additional surgical impact on the abdominal wall, and the visualisation of the preperitoneal space. However, the increasing number of different open techniques reflects the ongoing search by surgeons for the ideal approach to this type of surgery.

There is only one comparative randomised controlled study on open preperitoneal techniques. TIPP and TREPP techniques have been shown to be grossly comparable (fewer recurrences in the TIPP group are related to the learning curve) [30]. Other comparative studies between open preperitoneal techniques do not exist. More recent comparative studies between open preperitoneal and endoscopic techniques have shown either equivalent results [31–33] or, in some areas, better outcomes for open preperitoneal techniques [34].

A recent study compared open preperitoneal techniques with Shouldice and reported a better one-year-outcome for open preperitoneal techniques [35]. Open preperitoneal techniques may be a valuable alternative to the Lichtenstein technique for inguinal hernias. They seem to be associated with lower chronic pain, reduced opioid use and paraesthesia, and has benefits regarding patient-reported QoL [36, 37].

Scientific literature demonstrates that techniques such as TIPP and TREPP can be successfully performed as open preperitoneal procedures under local anaesthesia with analgosedation [23, 38]. In our view, this approach is feasible for all open preperitoneal techniques.

All open preperitoneal techniques can be done as day cases [39], making them suitable even in low-resource settings where laparo-endoscopic equipment is not available.

Open preperitoneal techniques are also suitable for recurrence procedures after anterior surgery with and without mesh [40]. Perhaps we must differentiate pure posterior (Ugahary and TREPP) and posterior approaches via the inguinal canal (TIPP and ONSTEP) as the latter is more difficult to realise after a previous anterior approach. The open posterior approach also appears to be feasible for complex inguinal hernias [41]. In our opinion, complex inguinal hernias are more dependent on the expertise of the surgeon. The MOPP technique seems to be effective for all primary groin hernias [19] and for primary scrotal hernias [20]. The authors believe that primary scrotal hernias can be successfully treated using the TIPP and TREPP techniques but are not ideal for the ONSTEP technique.

### Limitations

There is a lack of comparative randomised scientific studies between the different open preperitoneal techniques, as well as studies involving various patient groups, including long-term follow-ups.

Due to the limited scientific evidence, expert bias may influence the statements presented in this article.

## Conclusion

Over the past three decades, several new open preperitoneal techniques have been introduced for hernia repair. Despite their theoretical advantages, these techniques have not gained broad acceptance. Open preperitoneal approaches for groin hernia repair are straightforward and safe, often yielding results comparable to, or better than, other techniques [22]. Further standardisation of these methods is crucial for education and training purposes and for future comparative scientific studies.
